# American Dental Association

**Published:** 1891-04

**Authors:** 


### AMERICAN DENTAL ASSOCIATION.

Attention is called to the notice of the committee on another page. The matter of selection of a place for the meeting of the American Dental Association was left in their hands, and it will be held at Saratoga, New York.

While there can be no fault found with this decision, as the whole question has doubtless been well considered, it will unquestionably be felt by some that a more inland location would have been preferable. The expectation was that the committee would have selected Niagara Falls.

Now that the matter is settled, it is to be hoped that the societies will send full representations. This meeting should result in an effort to effect a change for the better in this organization. The profession needs a representative body.

